# Impact of phosphodiesterases PDE3 and PDE4 on 5-hydroxytryptamine receptor4-mediated increase of cAMP in human atrial fibrillation

**DOI:** 10.1007/s00210-020-01968-1

**Published:** 2020-09-19

**Authors:** Bernardo Dolce, Torsten Christ, Nefeli Grammatika Pavlidou, Yalin Yildirim, Hermann Reichenspurner, Thomas Eschenhagen, Viacheslav O. Nikolaev, Alberto J Kaumann, Cristina E Molina

**Affiliations:** 1 Department of Experimental Pharmacology and Toxicology, University Medical Center Hamburg-Eppendorf, Hamburg, Germany; 2 DZHK (German Center for Cardiovascular Research), Partner Site Hamburg/Kiel/Lübeck, Hamburg, Germany; 3 Institute of Experimental Cardiovascular Research, University Medical Center Hamburg-Eppendorf, Martinistrasse 52, W23, 20246 Hamburg, Germany; 4 Department of Cardiovascular Surgery, University Heart Center Hamburg, Hamburg, Germany; 5 Pharmacology, University of Murcia, Murcia, Spain

**Keywords:** 5-HT, Atrial fibrillation, cAMP, Human cardiomyocytes, Phosphodiesterases

## Abstract

Atrial fibrillation (AF)–associated remodeling includes contractile dysfunction whose reasons are only partially resolved. Serotonin (5-HT) increases contractile force and causes arrhythmias in atrial trabeculae from patients in sinus rhythm (SR). In persistent atrial fibrillation (peAF), the force responses to 5-HT are blunted and arrhythmic effects are abolished. Since force but not arrhythmic responses to 5-HT in peAF could be restored by PDE3 + PDE4 inhibition, we sought to perform real-time measurements of cAMP to understand whether peAF alters PDE3 + PDE4-mediated compartmentation of 5-HT_4_ receptor-cAMP responses. Isolated human atrial myocytes from patients in SR, with paroxysmal AF (paAF) or peAF, were adenovirally transduced to express the FRET-based cAMP sensor Epac1-camps. Forty-eight hours later, cAMP responses to 5-HT (100 μM) were measured in the absence or concomitant presence of the PDE3 inhibitor cilostamide (0.3 μM) and the PDE4 inhibitor rolipram (1 μM). We successfully established real-time cAMP imaging in AF myocytes. 5-HT increased cAMP in SR, paAF, and peAF, but in line with previous findings on contractility, this increase was considerably smaller in peAF than in SR or paAF. The maximal cAMP response to forskolin (10 μM) was preserved in all groups. The diminished cAMP response to 5-HT in peAF was recovered by preincubation with cilostamide + rolipram. We uncovered a significantly diminished cAMP response to 5-HT_4_ receptor stimulation which may explain the blunted 5-HT inotropic responses observed in peAF. Since both cAMP and force responses but not arrhythmic responses were recovered after concomitant inhibition of PDE3 + PDE4, they might be regulated in different subcellular microdomains.

**Electronic supplementary material:**

The online version of this article (10.1007/s00210-020-01968-1) contains supplementary material, which is available to authorized users.

## Introduction

Atrial fibrillation (AF) is the most frequent arrhythmia but its treatment remains a challenge. Conventional antiarrhythmic drugs have limited efficacy and increase the risk of ventricular arrhythmia (Nattel et al. 1994; Dobrev and Nattel 2010). Modest progress with anti-AF drugs is mostly due to the fact that compounds were produced in the absence of specific molecular targets. Refinement of conventional ion channel blocker therapy as an atrial-selective approach has not yet led to an improvement. Therefore, there is an urgent need to better understand the molecular pathophysiology of AF.

One important mechanism in the regulation of excitation-contraction coupling in cardiomyocytes is the phosphorylation of key proteins by activation of the cAMP and the cAMP-dependent protein kinase (PKA) signaling cascade. Phosphodiesterases (PDEs) are hydrolytic enzymes which degrade cAMP and limit the phosphorylation of PKA targets in specific myocyte compartments. PDEs comprise a large group of isoenzymes that are divided into 11 PDE families (Conti and Beavo 2007). Of these, PDE4 contributes most of the cAMP-hydrolytic activity in rodent ventricle (Rochais et al. 2006), while in human atria, cAMP is mainly hydrolyzed by PDE3 (Galindo-Tovar et al. 2009; Molina et al. 2012). Inhibition of either PDE3 or PDE4 leads to increase of the propensity of 5-HT-evoked arrhythmias in human atrial trabeculae from patients in sinus rhythm (SR) and with paroxysmal AF (paAF) but not with persistent AF (peAF) (Berk et al. 2016). Furthermore, it has been shown that the activity of PDE4 is reduced in peAF (Molina et al. 2012). FRET experiments in human atrial myocytes (HAMs) from patients in SR have identified PDE3 and to a smaller extent PDE4 to control both basal cAMP levels and cAMP responses under β-adrenergic stimulation (Molina et al. 2012).

5-HT increases force of contraction (Kaumann et al. 1990) and causes arrhythmias through 5-HT_4_ receptors in human atrial trabeculae from patients in SR (Kaumann 1994; Sanders and Kaumann 1994; Sanders et al. 1995; Pau et al. 2007). However, in peAF patients, positive inotropic effects of 5-HT were markedly reduced and 5-HT-induced arrhythmias were abolished (Christ et al. 2014). In SR and paAF, combined inhibition of PDE3 and PDE4 increased force responses of 5-HT as well as arrhythmic responses (Berk et al. 2016). In peAF, this treatment reversed the blunted force responses but did not restore arrhythmic responses to 5-HT (Berk et al. 2016). While in peAF force responses to 5-HT are mainly controlled by PDE3 (Berk et al. 2016), the abolished 5 HT-induced arrhythmias are not affected by PDE3 or PDE4, suggesting that force and arrhythmic responses may be regulated by distinct subcellular compartments.

Since none of these effects has been correlated with actual intracellular cAMP levels, we sought to establish an imaging approach for real-time monitoring of this crucial second messenger in primary HAMs from AF patients. To understand whether the blunted force responses or abolished arrhythmic responses to 5-HT in peAF result from a decrease of cAMP triggered by the 5-HT4 receptor stimulation, increased activity of PDEs or both, we established live cell imaging of cAMP and measured the effects of 5-HT and PDE inhibition on cAMP levels in HAMs from patients with SR, paAF, and peAF. Here, we demonstrate that cAMP responses to 5-HT are strongly reduced in peAF but they can be restored by concomitant PDE3 and PDE4 inhibition. Since similar effects have previously been shown for positive inotropic but not for arrhythmia responses to 5-HT, we suggest that these two effects might be regulated in different subcellular microdomains.

## Materials and methods

### Patients

Atrial tissue was obtained from patients undergoing cardiac surgery at the University Heart Center Hamburg. All patients gave written informed consent, according to the guidelines of the ethical review committee Hamburg, Germany. The study followed the declaration of Helsinki. Atrial tissue samples were obtained from a total of 30 patients undergoing cardiac surgery at the Department of Cardiovascular Surgery of the University Medical Center Hamburg-Eppendorf (UKE). Details regarding the clinical characteristics of the patients and their treatments are shown in Table 1.

**Table 1:** Details regarding the clinical characteristics of the patients and their treatments

	SR	paAF	peAF
Patients (*n*)	15	5	10
Male (*n*)	13 (86.7%)	4 (80.0%)	7 (70.0%)
Female (*n*)	2 (13.3%)	1 (20.0%)	3 (30.0%)
Age at surgery (years)	64.1	58.6	68.2
Indication for surgery			
Coronary artery disease (*n*)	11 (73.3%)	4 (80.0%)	2 (20.0%)
Valvular heart disease (*n*)	2 (13.3%)	1 (20.0%)	6 (50.0%)
Both (*n*)	2 (13.3%)	0 (0.0%)	1 (10.0%)
Dilated cardiomyopathy	0 (0.0%)	0 (0.0%)	1 (10.0%)
Cardiovascular comorbidity			
Arterial hypertension (*n*)	9 (60.0%)	3 (60.0%)	5 (50.0%)
Diabetes mellitus (*n*)	3 (20.0%)	0 (0.0%)	2 (20.0%)
Hyperlipoproteinemia (*n*)	2 (13.3%)	1 (20.0%)	2 (20.0%)
Echocardiography data			
LVEF (%)	56.0	52.3	54.5
Medication			
ACE inhibitor (*n*)	6 (40.0%)	4 (40.0%)	6 (60.0%)
AT1 receptor antagonist (*n*)	2 (13.3%)	0 (0.0%)	0 (0.0%)
Beta blocker (*n*)	4 (26.7%)	4 (80.0%)	5 (50.0%)
Ca^2+^ channel blocker (*n*)	2 (13.3%)	1 (20.0%)	1 (10.0%)
Diuretics (*n*)	2 (13.3%)	0 (0.0%)	3 (30.0%)
Digitalis (*n*)	0 (0.0%)	0 (0.0%)	0 (0.0%)
Nitrates (*n*)	0 (0.0%)	0 (0.0%)	0 (0.0%)

### Isolation, culture, and infection of human atrial myocytes

**Fig. 1:**
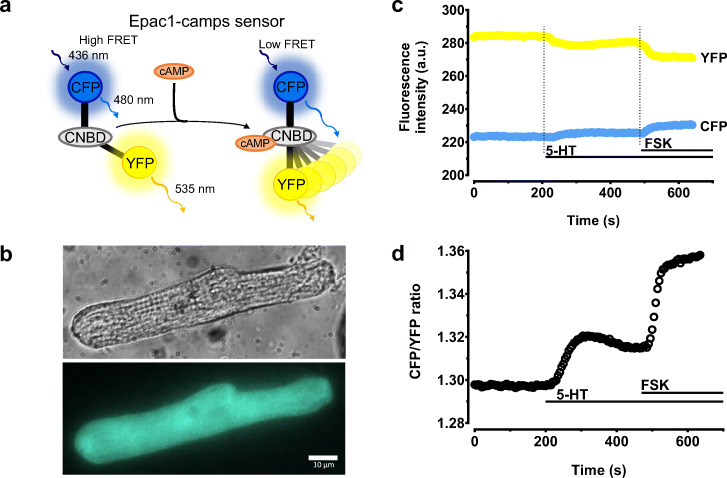
Increase of cAMP levels after stimulation of 5-HT receptors in human atrial myocytes. **a** Schematics of the Epac1-camps sensor containing yellow (YFP) and cyan fluorescent proteins (CFP) as donor and acceptor fluorophores linked to a cyclic nucleotide-binding domain (CNBD) of Epac1. Binding of cAMP to this domain leads to a conformational change resulting in a decrease of FRET (Nikolaev et. al 2004; Nikolaev and Lohse 2006). **b** Representative image of human AF atrial myocyte (HAM) after 48 h of culture and transfected with the cytosolic cAMP-sensor Epac1-camps (top, transmitted light image; bottom, fluorescence image in the CFP channel). **c** Real-time measurement of cAMP levels in this HAM. The graph shows the time course of cyan fluorescent protein (CFP) and yellow fluorescent protein (YFP) emissions in response to 5-HT (100 μM) and forskolin (+FSK, 10 μM). Increased cAMP binding to the sensor induces a decrease in fluorescence resonance energy transfer between CFP (excited at 440 ± 20 nm) and YFP visualized by a simultaneous increase in donor emission (CFP; blue squares) and a decrease in acceptor emission (YFP; yellow squares). Acquisitions were performed every 5 s. The corresponding CFP/YFP ratio trace is shown in **d** increase in the CFP/YFP ratio is indicative of an increase in intracellular cAMP

Tissues from atrial appendages were immediately transferred to Custodiol® solution (Dr. Franz Köhler Chemie GmbH, Bensheim, Germany) and used for cell isolations within 10–20 min. Atrial cardiomyocytes were isolated by enzymatic digestion as previously described (1–3). Briefly, tissue was cut into small pieces and incubated at 36 °C in a Ca^2+^-free solution containing 0.5 mg ml^−1^ collagenase (Worthington type 1, 240 U mg^−1^; Lakewood, New Jersey, USA), 0.5 mg ml^−1^ proteinase (Sigma type XXIV, 11 U mg^−1^; St. Louis, Missouri, USA) and 2% bovine serum albumin (BSA; Sigma, St. Louis, Missouri, USA). After 30 min, the tissue was moved from the enzymatic solution to a Ca^2+^-free solution containing 2 μM blebbistatin (Sigma, St. Louis, Missouri, USA) and 5% BSA and the tissue pieces left were disaggregated with a Pasteur pipette. The remaining tissue was digested for 3 × 15 min in a fresh Ca^2+^-free solution containing 0.4 mg ml^−1^ of the mentioned above collagenase and 2% BSA. Freshly isolated cells were suspended in minimal essential medium (MEM: M 4780; Sigma, St. Louis, Missouri, USA), 2.5% fetal bovine serum (FBS, Invitrogen, Cergy-Pontoise, France), 1% penicillin-streptomycin, 2% HEPES (pH 7.6), and 2 μM blebbistatin. Cells were then plated on 35 mm laminin-coated culture dishes (10 μg ml^−1^ laminin; Sigma, St. Louis, Missouri, USA). After 2 h, the medium was replaced by 2 ml per dish of FBS-free MEM (Gibco/Invitrogen, Cergy-Pontoise, France) containing adenovirus encoding Epac1-camps (at the multiplicity of infection equal 200 plaque forming units per cell) (6). This sensor was used before in rat ventricular but not in human atrial myocytes (Bastug-Özel et al. 2019). Only striated rod-shaped myocytes were used for experiments (Fig. 1).

### Confocal imaging

Confocal microscopy was performed using Zeiss LSM 810 NLO microscope (Carl Zeiss MicroImaging, Jena, Germany) equipped with a Plan-Apochromat ×63/1.40 oil immersion objective. Cells were sequentially excited with a 405 nm laser for CFP and 488 nm laser for YFP and the emission was measured at 475/30 nm and 527/40 nm, for CFP and YFP respectively.

### Live cell imaging of intracellular cAMP

Förster resonance energy transfer (FRET) measurements were performed on myocytes 48 h after transduction with the cAMP-based sensor Epac1-camps (Nikolaev et al. 2004; Borner et al. 2011). Cytosolic cAMP was measured in a total of 64 isolated human atrial myocytes (HAMs). During FRET measurements, cells were maintained in a K^+^-Ringer solution containing (in mM) NaCl 121.6, KCl 5.4, MgCl_2_ 1.8; CaCl_2_ 1.8; NaHCO_3_ 4, NaH_2_PO_4_ 0.8, D-glucose 5, sodium pyruvate 5, HEPES 10, adjusted to pH 7.4 with NaOH. Images in yellow (YFP) and cyan fluorescent protein (CFP) emission channels were captured every 5 s using an inverted fluorescent microscope (Leica DMI3000 B) equipped with a 63× oil immersion objective, a beam-splitter Dual-View DV2 with a 505dcxr dichroic mirror, D480/30 m and D535/40 m emission filters (Photometrics), and a CMOS Camera (QIMAGING optiMOS). The FRET donor CFP was excited at 440 nm using a CoolLED light source. The exposure time was 5 ms. Signals were analyzed offline by the software Micro-Manager 1.4.5 together with ImageJ (Sprenger et al. 2012; Schotten et al. 2001). Excel and Prism5.0 were used to calculate the corrected FRET ratio and for statistical analysis. All experiments were performed at room temperature.

### Drugs

5-Hydroxytryptamine (5-HT) hydrochloride, forskolin (FSK), rolipram (Rol), and cilostamide (Cil) were purchased from Sigma. Rolipram and cilostamide were solubilized in dimethyl sulfoxide and in K^+^-Ringer solution. The final concentration of dimethyl sulfoxide was less than 0.1%, which by itself did not affect cAMP levels. All the drugs were dissolved in a K^+^-Ringer solution previously described reaching the final concentration of 100 μM 5-HT, 1 μM rolipram, and 300 nM cilostamide.

### Statistics

Values are expressed as mean ± SEM. D’Agostino and Pearson omnibus normality test was used to test for normal distribution. If normally distributed, statistical significance was evaluated using a paired Student’s *t* test or one-way ANOVA followed Tukey’s multiple comparisons post hoc test when more than two groups were compared. In case of skewed distribution, data were analyzed by non-parametric Mann-Whitney test when two groups or by non-parametric Kruskal-Wallis test when more than two groups were compared. Differences were considered statistically significant when *p* < 0.05. All statistical tests were done with GraphPad Prism 6.0 (GraphPad Software, San Diego, USA).

## Results

### Real-time monitoring of cAMP in HAMs

To perform real-time cAMP measurements in HAMs, we cultured isolated myocytes for 48 h and transduced them with an adenovirus to express the FRET-based cAMP biosensor Epac1-camps (Fig. 1a) (Nikolaev and Lohse 2006). Although we previously used this protocol for SR cells and a similar biosensor for cytosolic cAMP called Epac2-camps (Molina et al. 2012), it has not been applied to AF cells so far. Even after 2 days in culture, HAMs isolated from AF patients and expressing the biosensor still showed a normal morphology and membrane structure (Fig. 1b). They also responded to 5-HT and FSK application with a change of FRET (Fig. 1c, d). Basal FRET ratios were similar in all studied groups, although paAF and specially peAF myocytes showed a tendency towards increased basal FRET signal which might indicate a slightly higher basal cAMP levels in AF (Supplemental Fig. 1a). Importantly, no obvious differences in the subcellular sensor localization could be observed between SR and AF cells by confocal microscopy (Supplemental Fig. 1b).

### Reduced 5-HT triggered cAMP levels in peAF

**Fig. 2:**
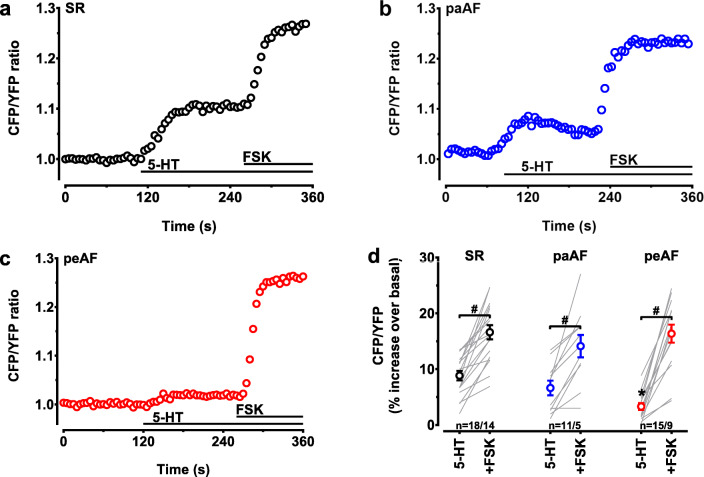
cAMP responses to 5-HT but not to FSK are reduced in peAF. Time courses of representative FRET signals indicating changes in cytosolic cAMP in 3 HAMs from patients with SR (**a**), paAF (**b**), and peAF (**c**) exposed to 100 μM 5-HT and in the continuous presence of 5-HT to 10 μM forskolin (FSK). **d** Summary of the results from panels **a**–**c**. Depicted are FRET responses to both 5-HT and forskolin in the presence of 5-HT (+FSK) in individual myocytes. Mean values ± SEM are indicated by the circles. *n* = number of myocytes/number of patients. *p < 0.05 vs. 5-HT in SR (one-way ANOVA; Tukey’s multiple comparisons test, based on myocytes); ^#^*p* < 0.05 FSK vs. 5-HT (paired *t* test). Values for FSK were not significantly different between groups (one-way ANOVA; Tukey’s multiple comparisons test)

We first investigated whether the reduced inotropic effect of 5-HT in peAF could be due to a reduced increase in cAMP. Myocytes were stimulated with saturating concentrations of 5-HT, and 10 μM FSK was added in the presence of 5-HT at the end of each experiment to measure the maximal cAMP-generating capacity (as in Fig. 1). Typical time courses of the FRET signals are shown in Figs. 2 a–c. 5-HT (100 μM) increased cytosolic cAMP levels and FSK (10 μM) increased cAMP even further in SR, paAF, and peAF. However, cAMP responses to 5-HT alone were 63% smaller in peAF than in SR (Fig. 2d), suggesting either a diminished ability of the 5-HT_4_ receptor to activate adenylyl cyclase or diminished ability of adenylyl cyclase to produce cAMP. However, in paAF cells, 5-HT/cAMP responses were comparable with those of SR (Fig. 2a–d). The FSK effects in the presence of 5-HT were not different between the three groups, demonstrating intact ability of adenylyl cyclase to produce cAMP in myocytes from patients with peAF.

### PDE3 and PDE4 inhibition restores the 5-HT-triggered increase of cAMP in peAF

**Fig. 3:**
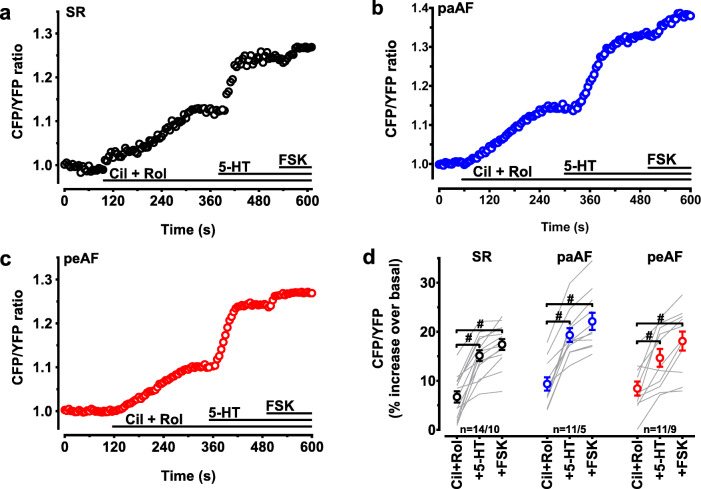
Inhibition of PDE3 and PDE4 restores cAMP response to 5-HT in peAF. Time courses of the representative cAMP responses in 3 HAMs from patients in SR (**a**), paAF (**b**), and in peAF (**c**) exposed to 0.3 μM cilostamide and 1 μM rolipram (Cil + Rol). Summary of the results is in panel. **d** Depicted are FRET responses to Cil + Rol, to 100 μM 5-HT and to 10 μM forskolin in the presence of Cil + Rol and 100 μM 5-HT (+FSK) in individual myocytes. Mean values ± SEM are indicated by the circles. *n* = number of myocytes/number of patients. ^#^*p* < 0.05 vs. the corresponding values for Cil + Rol alone. Values for FSK in the presence of Cil + Rol and 5-HT were not significantly different between groups (one-way ANOVA followed by Tukey’s multiple comparisons test)

**Fig. 4:**
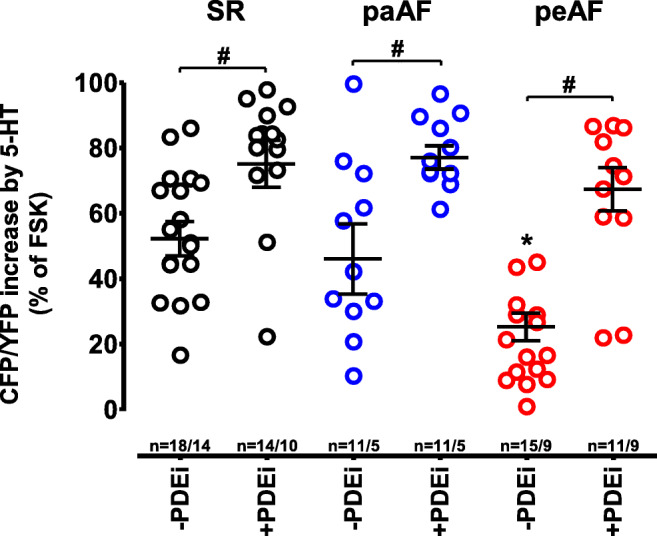
Inhibition of PDE3 and PDE4 increases cAMP levels upon 5-HT stimulation in peAF. Analysis of 5-HT responses in the absence (−PDEi) and in the presence of PDE inhibitors (0.3 μM cilostamide and 1 μM rolipram, +PDEi), indicating single-value cells based and mean values ± SEM patients based. *n* = number of myocytes/number of patients. *p < 0.05 vs. in the absence of PDE(−PDEi) in SR or paAF (one-way ANOVA, followed by Tukey’s multiple comparisons test); #*p* < 0.05 vs. respective values for −PDEi (non-parametric Mann-Whitney test)

Increased PDE activity could result in reduced cAMP responses upon 5-HT_4_ receptor activation. To test this possibility, we measured cAMP levels under concomitant cilostamide (Cil, 0.3 μM) and rolipram (Rol, 1 μM) treatment of HAM from the three groups of patients. Typical time courses are shown in Fig. 3 a–c. Interestingly, the relative increase of cytosolic cAMP caused by the two PDE inhibitors was not greater in peAF than in SR or paAF (Fig. 3d, *p* = 0.34, one-way ANOVA), refuting the hypothesis that a higher PDE activity per se might reduce the cAMP increase after 5-HT_4_ receptor stimulation in peAF. Next, we calculated the effect of concomitant PDE3 and PDE4 inhibition on 5-HT-evoked cAMP accumulation measured in Fig. 3 a–c. The magnitude of the 5-HT-induced cAMP levels after Cil + Rol preincubation was no longer smaller in peAF than in SR and paAF (Figs. 3 and 4), and the further increase by FSK (10 μM) was not statistically significant between the groups (Fig. 3).

## Discussion

Classical antiarrhythmic drugs were developed without specific molecular targets and there is an urgent need to better understand AF molecular pathophysiology. We performed this study to establish live cell imaging of cAMP in AF myocytes and used this approach to understand the molecular mechanisms of 5-HT/cAMP response and its regulation by PDEs in paAF and peAF.

5-HT caused a smaller increase of cAMP in HAMs from peAF patients than from SR patients and paAF patients. This is in agreement with the depressed force response to 5-HT in trabeculae from peAF patients (Christ et al. 2014). Inhibition of cAMP hydrolysis with the concomitant administration of the PDE3-selective inhibitor cilostamide (0.3 μM) and the PDE4-selective inhibitor rolipram (1 μM) restored cAMP levels in peAF patients. This agrees with the recovery of the force response after treatment with the two PDE inhibitors (Berk et al. 2016). In contrast, concomitant application of cilostamide and rolipram failed to restore the arrhythmic responses to 5-HT in peAF. Of interest, either cilostamide or rolipram caused only marginal increases of 5-HT-induced arrhythmias in trabeculae of SR patients. However, cilostamide and rolipram administered together enhanced four-fold the 5-HT-induced arrhythmias in trabeculae from SR patients but not at all in trabeculae from peAF patients (Berk et al. 2016), despite the robust increases in cAMP in myocytes from both SR and peAF (Figs. 3 and 4). It could be proposed that the subcellular microdomain through which PDEs enhance 5-HT-induced arrhythmias is disrupted in peAF.

The reduced cAMP responses to 5-HT in peAF are associated with preserved I_Ca,L_ responses but blunted force (Berk et al. 2016). In HAM from patients with peAF, cAMP responses by 5-HT were reduced by 63% while increases by FSK were preserved. However, it should be noted that despite the preserved cAMP responses to FSK in peAF (Fig. 3), the increase of I_Ca,L_ with FSK in peAF was not larger than with 5-HT (Christ et al. 2014). This finding indicates that even if the cytosolic cAMP response upon 5-HT is reduced in peAF, it is possible to activate I_Ca,L_ maximally compared with FSK. In contrast to virtually maximum I_Ca,L_ increases observed with 5-HT and FSK, the 5-HT-induced FRET responses were reduced by 63%, and force responses to 5-HT were reduced by 85% in peAF (Berk et al. 2016; Christ et al. 2014). We suggest that the preserved effects of 5-HT on I_Ca,L_ in peAF are due to restricted pool of cAMP within the L-type Ca^2+^ channel microdomain. However, cytosolic cAMP levels strongly affect various intracellular targets phosphorylated by PKA in distinct subcellular microdomains to control contractile force and relaxation upon 5-HT stimulation. As an alternative to the subcellular compartmentation, the putative mechanism mediating 5-HT-induced arrhythmias, which are significantly reduced in peAF, could be independent of cAMP, PDE3, and PDE4. Nevertheless, arrhythmias in human atrial trabeculae evoked by 5-HT can be abolished by 5-HT receptor antagonists (Kaumann 1994). 5-HT induces arrhythmias in mice only when the 5-HT receptor is overexpressed (Gergs et al. 2010). Both findings argue against receptor-independent arrhythmia induction by 5-HT. More importantly, application of an inhibitor of PKA stops 5-HT-induced arrhythmias in atria from mice transgenic for the 5-HT receptor (Gergs et al. 2013).

In the future, it will be important to study cAMP response in distinct subcellular microdomains using targeted cAMP biosensors. For example, microdomains which are important for Ca^2+^ handling and contractility (i.e., L-type Ca^2+^ channel, SERCA2A, ryanodine receptors, or troponin I-associated microdomains) can be analyzed using recently described targeted biosensors (Surdo et al. 2017) to dissect the molecular mechanisms of the compartmentalized 5-HT/cAMP responses.

## Conclusions

We conclude that there are profound differences in the cAMP regulation by PDEs in peAF compared with SR or paAF. Effects of PDE inhibition on 5-HT-evoked cAMP and force response are increased in peAF while 5-HT-mediated arrhythmogenic effects are not restored by PDE inhibition. Taking together, our results suggest that PDE3 and PDE4 control cytosolic 5-HT/cAMP-stimulated inotropic responses, whereas arrhythmic responses may be regulated by another pool of cAMP which is compartmentalized in a distinct subcellular microdomain.

## Limitations

The conclusion that 5-HT triggers less cytosolic cAMP in peAF is only valid as long as the affinity of 5-HT to its receptor(s) is comparable between different groups and 100 μM of 5-HT would lead to full occupancy of 5-HT_4_ receptors. Although there are no radioligand binding data available for 5-HT receptors in peAF, from concentration-response dependencies for 5-HT on force, it can be concluded that 100 μM 5-HT used in our study is indeed a saturating agonist concentration for both in SR and peAF (Berk et al. 2016). Regarding the extent of PDE inhibition, similar to our former work (Berk et al. 2016), we used 0.3 μM cilostamide to inhibit PDE3 and 1 μM rolipram to inhibit PDE4. These rather low concentrations were chosen to avoid cross-reactivity by the inhibitors. It should be noted that the actual extent of inhibition of PDE3 and PDE4 is expected to be different with less inhibition of PDE4 by 1 μM rolipram than inhibition of PDE3 by 0.3 μM cilostamide. As a result, effects of concomitant inhibition of PDE3 and PDE4 demonstrated here may depend more on PDE3 than on PDE4. We are aware of that limitation. However, these concentrations were chosen on purpose to directly compare cAMP data with our previously published results on force and arrhythmias.

## Electronic supplementary material

ESM 1 (XLSX 23 kb)

Supplemental Figure 1 Basal FRET ratios and sensor localization in Epac1-camps expressing human atrial myocytes, (a) Comparison of the basal FRET ratios in myocytes from the three groups of patients (SR. paAF, peAF) (b) Representative confocal images (*n* > 5) of HAMs from patients in SR (top) and peAF (bottom) expressing Epac1-sensor. No obvious differences in the subcellular sensor localization could be observed between SR and AF. (PDF 157 kb)
