# Supplementary figures and images for: Impact of phosphodiesterases PDE3 and PDE4 on 5-hydroxytryptamine receptor4-mediated increase of cAMP in human atrial fibrillation
Source: Naunyn Schmiedebergs Arch Pharmacol. 2020 Sep 19;394(2):291–8. doi: 10.1007/s00210-020-01968-1 (PMC7835186; doi:10.1007/s00210-020-01968-1)

**a**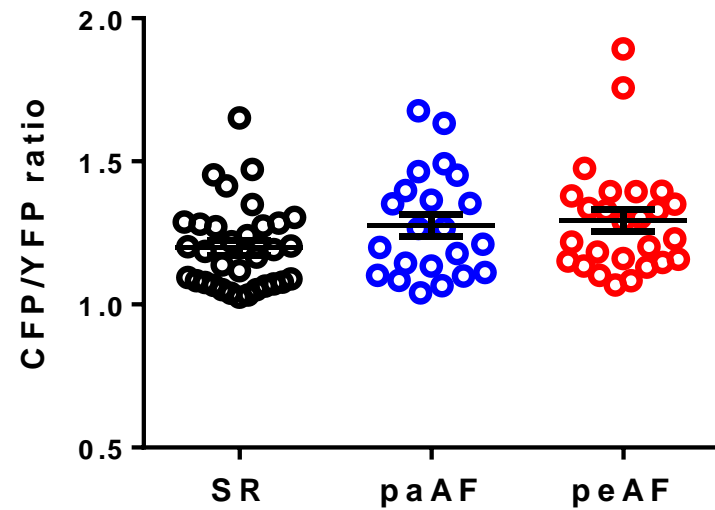**b**

YFP

CFP

Overlay

SR

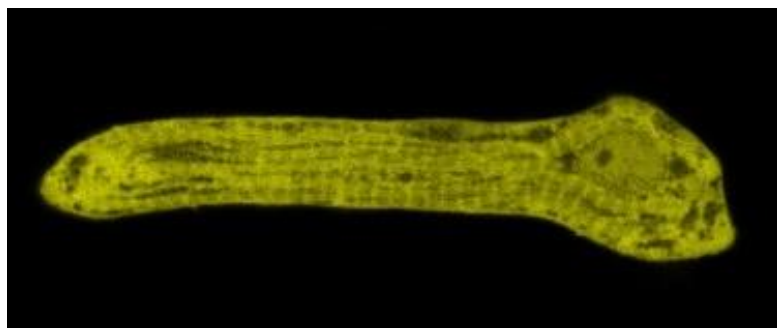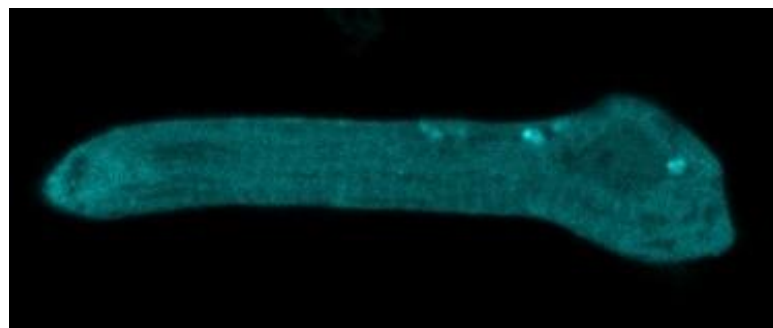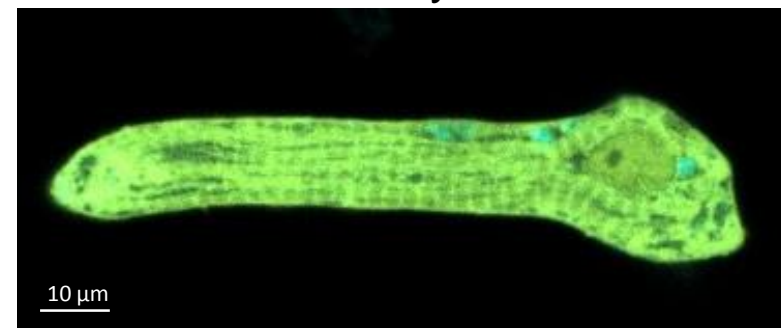

AF

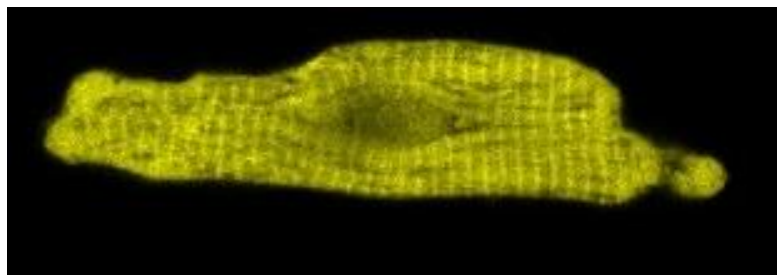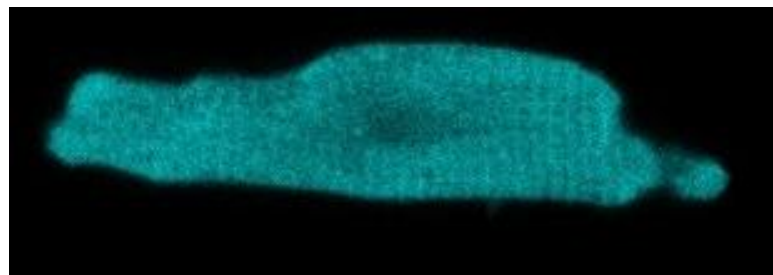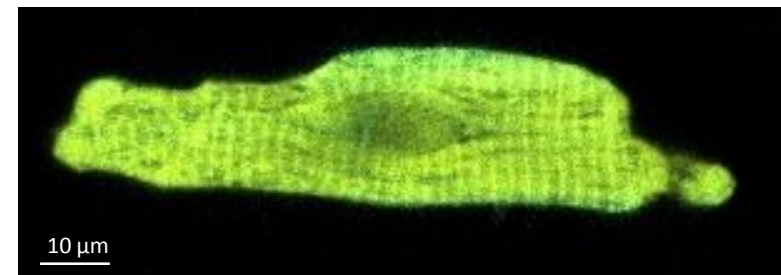**Supplemental Figure 1**

Supplement: Supplementary file 2 — Basal FRET ratios and sensor localization in Epac1-camps expressing human atrial myocytes, (a) Comparison of the basal FRET ratios in myocytes from the three groups of patients (SR. paAF, peAF) (b) Representative confocal images (n > 5) of HAMs from patients in SR (top) and peAF (bottom) expressing Epac1-sensor. No obvious differences in the subcellular sensor localization could be observed between SR and AF. (PDF 157 kb) [file 210_2020_1968_MOESM2_ESM.pdf]
